# Metabolic insights into the warfarin-mango interaction: A pilot study integrating clinical observations and metabolomics

**DOI:** 10.5599/admet.2740

**Published:** 2025-06-08

**Authors:** Piyapat Rattanasuwan, Prem Lertpongpipat, Natthapat Hiranchatchawal, Konwalin Wannaphueak, Sakonwan Pounghom, Parinya Thongkhao-on, Matchuda Suwanthai, Duangthip Sompradee, Auiporn Saithongdee, Churdsak Jaikang, Preechaya Tajai

**Affiliations:** 1 Faculty of Medicine, Chiang Mai University, Chiang Mai 50200, Thailand; 2 Hua Hin Hospital, Prachuap Khiri Khan 77110, Thailand; 3 Department of Forensic Medicine, Faculty of Medicine, Chiang Mai University, Chiang Mai, 50200, Thailand; 4 Metabolomic Research Group for Forensic Medicine and Toxicology, Department of Forensic Medicine, Faculty of Medicine, Chiang Mai University, Chiang Mai 50200, Thailand

**Keywords:** Biomarker, drug interaction, ^1^H-NMR-based metabolomics, metabolites, personalized anticoagulant

## Abstract

**Background and purpose:**

Warfarin is a widely prescribed oral anticoagulant for the prevention and treatment of thromboembolic events, frequently used in patients with atrial fibrillation. However, its effectiveness is often challenged by a narrow therapeutic range and significant inter-patient variability in dosage requirements and treatment responses. Drug interactions remain a critical concern, as they heighten the risk of supratherapeutic anticoagulation. Reports of interactions between warfarin and mango have documented cases of elevated international normalized ratio (INR) following mango consumption, although the underlying molecular mechanisms remain unclear.

**Experimental approach:**

This study investigates the molecular basis of the warfarin-mango interaction using proton nuclear magnetic resonance (^1^H-NMR)-based metabolomics. In a pre-post design study, plasma samples were collected from patients on long-term warfarin therapy (>6 months) who exhibited supratherapeutic INR levels after consuming mango. After a two-week discontinuation of mango consumption, additional plasma samples were collected once INR levels returned to the therapeutic range.

**Key results and conclusion:**

This is the first study to utilize ^1^H-NMR metabolomics to explore warfarin-mango interactions, integrating clinical observations with metabolic insights. Findings suggest that a reduction in glycerol 3-phosphate may impair glycolysis, disrupting platelet activation and contributing to the elevated INR levels observed in all patients. These results underscore the potential for ^1^H-NMR metabolomics to elucidate drug-food interactions, advancing personalized anticoagulant management and improving patient safety.

## Introduction

**Figure 1.**
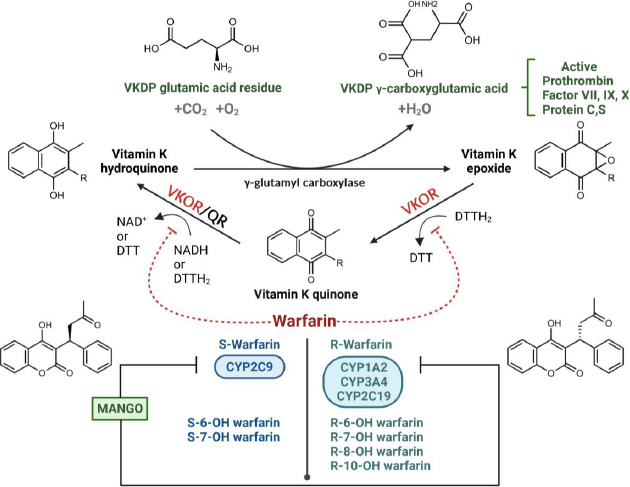
Summary of the warfarin-mango interaction mechanism in the context of vitamin K metabolism and warfarin metabolism. Abbreviations used: DTT (Dithiothreitol); QR (Quinone reductase); VKDP (Vitamin K-dependent protein); VKOR (Vitamin K epoxide reductase)

Warfarin remains a widely prescribed oral anticoagulant for the prevention and treatment of thromboembolism events, frequently used in patients with atrial fibrillation (AF) [1,2]. However, its effectiveness is challenged by a narrow therapeutic range and significant variability in dosage requirements and treatment responses among patients [1]. The emergence of new effective, safe anticoagulants, specifically non-vitamin K antagonist oral anticoagulants (NOACs), has resulted in their recommendation in international guidelines [1,2]. Nonetheless, NOACs still present challenges in terms of cost-effectiveness, particularly in resource-limited healthcare settings, including community hospitals in Thailand [2,3]. The primary complication regarding treatment lies in the risk of supratherapeutic anticoagulation, which can arise as a consequence of various factors [2,4]. Genetic factors and drug interactions predominantly contribute to this complication risk [4-8]. Warfarin disrupts the conversion of vitamin K and its epoxide form, which is essential for the carboxylation of glutamate residues into γ-carboxyglutamates (Gla) on vitamin K-dependent proteins, including coagulation factors II, VII, IX, and X [9]. This γ-carboxylation process requires the reduced form of vitamin K, known as vitamin K hydroquinone (vitamin KH_2_), and facilitates the binding of these coagulation factors to phospholipid surfaces, thereby enhancing blood coagulation [9]. Warfarin inhibits the synthesis of vitamin KH_2_ by targeting the enzyme vitamin K epoxide reductase complex 1 (VKORC1), thus limiting the γ-carboxylation of vitamin K-dependent coagulation proteins (Figure 1) [9]. Impaired clearance of warfarin results in elevated plasma concentrations, as indicated by an increase in international normalized ratio (INR) [4,7,9]. Such disruptions may lead to severe adverse effects, including an elevated risk of bleeding and significant haemorrhage [4,7,9]. Warfarin is a racemic mixture composed of R- and S-enantiomers, with their metabolism predominantly mediated by cytochrome P450 (CYP) enzymes, including CYP2C9, CYP2C19, CYP1A2 and CYP3A4 (Figure 1) [4,6,7]. S-warfarin, the more potent enantiomer, is primarily metabolized by CYP2C9 into 6- and 7-hydroxywarfarin [4,7,10]. In contrast, R-warfarin undergoes metabolism via CYP1A2 to form 6- and 8-hydroxywarfarin, and by CYP3A4 to produce 10-hydroxywarfarin [4,7,10]. Additionally, R-warfarin is metabolized by carbonyl reductases into diastereoisomeric alcohols [4,7,10]. CYP2C19 is involved in the conversion of both R- and S-warfarin into 6-, 7-, and 8-hydroxywarfarin, though it plays a more significant role in the hepatic metabolism of R-warfarin, with minimal impact on S-warfarin [4,7,10].

Dietary supplements, herbs, and foods can interfere with the pharmacological actions of warfarin through two principal mechanisms: by altering the pharmacokinetics (absorption, distribution, or metabolism) or by affecting the pharmacodynamics (the pharmacological effects resulting from interactions with other substances) [7,11]. The most prevalent cause of these interactions involves the inhibition or induction of the CYP2C9 and CYP3A4, which play a key role in the metabolism of warfarin [7]. The mango (*Mangifera indica*; Anacardiaceae family), originating from southern Asia, can also be found in Thailand [5,6]. Mango fruit is popular due to its sweet taste, but is also advantageous with regard to diet due to its richness in vitamins A, C and fibre [5]. However, previous studies have reported a potential interaction between mango fruit and warfarin based on real case practice [5,6]. One case report described problems in patients who experienced supratherapeutic INR levels after consuming mangoes daily for up to 1 month before their regular anticoagulation clinic follow-up, with no additional factors contributing to the elevated INR. After a 2-week follow-up during which mango ingestion ceased, the average INR decreased by 17.7 %, returning to the therapeutic range. Subsequent rechallenges involving mango fruit in two patients yielded consistent results, suggesting a correlation between mango consumption and supratherapeutic anticoagulation [5,7]. A medium-sized mango (130 g without seed) contains significant vitamin content, especially vitamin A (8061 IU) [5]. Several studies suggest that mango contains various bioactive compounds, including retinol and polyphenols [5,7,12]. There are reports suggesting that concurrent administration of warfarin and vitamin A may lead to an increased anticoagulant effect, particularly in the case of high doses of vitamin A [5]. Additionally, an *in vitro* study has shown that retinol strongly inhibits human CYP2C9 and CYP2C19, potentially leading to increased warfarin concentration [5-7,13,14]. Polyphenols in mango also interact with intestinal and hepatic enzyme systems, particularly by inhibiting CYP3A4 and CYP2D6 [5,6,14,15]. Additionally, mango may affect platelet aggregation, potentially complicating the anticoagulant effects of warfarin [6,7]. The interaction between warfarin and mango was determined using the Naranjo probability scale, results indicating it as “probable”, suggesting that mango ingestion might contribute to supratherapeutic anticoagulation [5]. Nevertheless, the molecular mechanism behind this interaction remains unexplained. This study aims to understand the interaction between warfarin and mango further, bridging the gap from clinical practice to molecular mechanisms through metabolomic analysis using proton nuclear magnetic resonance (^1^H-NMR).

## Experimental

### Reagents

Methanol, chloroform, deuterium oxide (D_2_O), and trimethylsilyl propanoic acid (TSP) were purchased from Sigma-Aldrich St. Louis, MO, USA. All chemicals used were of analytical grade.

### Ethical approval and informed consent

The study was conducted in line with the Declaration of Helsinki and was approved by the Institutional Review Board (Human Ethics Committee) of the Faculty of Medicine, Chiang Mai University, Thailand. The ethical approval reference number is No. 201/2023 (Study code: FOR-2566-0144). Eligible patients were those who had been taking warfarin continuously for more than 6 months and had experienced a warfarin-mango interaction, resulting in their INR being out of the therapeutic range during the study period. Before enrolment, written informed consent was acquired from all patients, and the study results were reported anonymously.

### Study design, setting and population

This was a pre-post study design conducted at a tertiary care hospital in Thailand. For the pre-study data, patients aged 18-70 years who had been continuously taking warfarin for more than 6 months and had experienced warfarin-mango interaction were recruited. Their monitored INR levels were supratherapeutic. Patients taking other anticoagulants, those with acute or chronic infections, and patients evaluated by pharmacists as non-compliant were excluded from this study. The post-study data were recorded after patients discontinued mango consumption and returned for a follow-up period of at least two weeks. During this period, their INR levels decreased to within the therapeutic range. A specific sample size was not calculated for the study, given the rarity of the incidence of warfarin-mango interaction. All patients who met the inclusion criteria and were identified during the study period were considered eligible.

### Sample preparation

Plasma samples were combined with an equal volume of acetonitrile and mixed for 10 min. Subsequently, the mixture was centrifuged at 4000 RPM for 10 min, and the supernatant was collected and lyophilized. A 0.6 mL solution containing 0.1 M TSP in D_2_O was then prepared. Finally, metabolite levels were quantified using 500 MHz NMR, employing a method specifically designed to mitigate interference from water resonance.

### Acquisition parameters

The proton NMR spectra were acquired using a Bruker AVANCE 500 MHz spectrometer (Bruker, Bremen, Germany), equipped with a Carr-Purcell-Meiboom-Gill (CPMG, RD 90°, (*t* 180°), *n* - acquire) pulse sequence for ^1^H-NMR measurements. The spectra were recorded at 27 °C with water suppression via pre-saturation. The experimental parameters included 16 scans, a 1-second relaxation delay, a 3.95-second acquisition time, an 8278.146 Hz spectral window, a 0.126 Hz free induction decay (FID) resolution, and a 60.40 dwell time (DW). A 90° pulse with 16 signal averages (NSAs) was applied. Baseline and phase corrections were conducted using TopSpin 4.0.7 software [16]. The spectra, recorded over a chemical shift range of 0 to 12 ppm and normalized to the total integrated area, were analysed for metabolite identification. Metabolite resonances were identified through human databases [17]. TSP was employed as an internal standard, enabling the quantification of 24 energy-related metabolites across all samples.

### Internal standard

TSP was selected as the internal standard (IS) based on its specific chemical properties, where all 14 protons are equivalent. This homogeneity guarantees that the TSP signal consistently appears at 0 ppm at 500 MHz and originates from a region with higher magnetic field intensity compared to other protons. A further advantage is that TSP is chemically inert and has a low boiling point in organic solvents, facilitating its efficient extraction from samples.

### Quality control

Distinct protocols were implemented for the quality control (QC) samples on the ^1^H-NMR platforms. These protocols were important for calibrating the system before, during, and after the analysis, ensuring consistent results and minimizing analytical variations. The QC samples were prepared by combining and thoroughly mixing equal amounts from each sample. These samples underwent the same procedural steps as the test samples, adhering to the previously established methods. An analysis of non-targeted metabolites was conducted using the procedures described.

### Peak assignment, chemical identification and ^1^H-NMR data analysis

Chemical structures were identified using the Human Metabolome Database [18] and referenced from a previous study [16,18]. Peak acquisition and *J*-coupling values were analysed utilizing Bruker TopSpin version 4.0.7 software. NMR spectra interpretation was primarily based on chemical shift values, essential for signal identification, integration, spin–spin coupling analysis, signal pattern examination, and coupling constant assessment. Each peak of non-targeted metabolites was carefully adjusted to ensure deviations of less than 0.01 ppm relative to the HMDB database.

For data export and spectrum visualization, MestRenova software (version 12.0.0, MestreLab Research, Santiago de Compostela, Spain) was employed [16,19]. Data were presented using median values, and normality was evaluated using the Kolmogorov–Smirnov test [16,19].

### Bioinformatics and statistical analysis

MetaboAnalyst [20] was employed to analyze differences in plasma metabolites during the warfarin-mango interaction and after mango discontinuation (control). Pathway enrichment and analysis were conducted using MetaboAnalyst, in combination with Metascape [21] which was utilized for the construction of metabolite-metabolite networks. MetaboAnalyst version 6.0 was also used to evaluate alterations in metabolic profiles during the warfarin-mango interaction through heatmaps, along with Pearson correlation coefficient calculations to explore metabolic changes. Key metabolites identified as potential biomarkers were further assessed via univariate receiver operating characteristic (ROC) curve analyses, with 95 % confidence intervals calculated using MetaboAnalyst version 6.0 for individual biomarkers. ROC curve analyses were also performed to evaluate the area under the curve (AUC) for INR values in comparison to glycerol 3-phosphate concentrations during the warfarin-mango interaction. These analyses were conducted by GraphPad Prism, version 8.3.0 for Windows [22].

Descriptive statistics were used to calculate the demographic characteristics of the 15 patients. The data are presented as the number (%) of patients and the mean ± standard deviation (SD). Statistical analysis, using a Wilcoxon signed-rank test to compare the difference between INR levels during mango ingestion and after discontinuation, was performed with GraphPad Prism version 8.3.0 for Windows [22].

## Results

### Demographic characteristics of patients

The fifteen eligible cases included 5 males (33.33 %) and 10 females (66.67 %). The underlying conditions indicating the need for warfarin therapy included atrial fibrillation (AF) in 12 patients (80.00 %) and mitral valve replacement (MVR) in 3 patients (20.00 %). All patients experienced an increase in INR following mango consumption, with reported intake ranging from 1 to 3 mangoes per day over a period of 5 days to 1 month prior to the observed drug interaction. Other factors that could contribute to an increase in INR, such as non-compliance, were not recorded. After mango fruit was identified as a possible cause of the supratherapeutic INR, patients were instructed to discontinue mango consumption. The average INR decreased from 4.57 ± 1.46 to 2.45 ± 0.39, the difference between INR levels during mango ingestion and after discontinuation being statistically significant (*p* < 0.0001), suggesting that mango intake may have influenced warfarin metabolism (Table 1).

**Table 1.** Patient characteristics

Variable	Warfarin-mango interaction	Following mango discontinuation*
	Mean ± SD (range)	
International normalized ratio	4.57 ± 1.46** (3.17-8.67)	2.45 ± 0.39 (1.73-3.10)
Underlying disease requiring warfarin therapy	*n* (Share, %)	
Atrial fibrillation	12 (80.00)	
Mitral valve replacement	3 (20.00)	
Gender	*n* (Share, %)	
Male	5 (33.33)	
Female	10 (66.67)	

*Control

**Wilcoxon signed-rank test (*p* < 0.0001)

### Determination of differences in plasma metabolites between warfarin interaction and control using ^1^H-NMR

**Figure 2.**
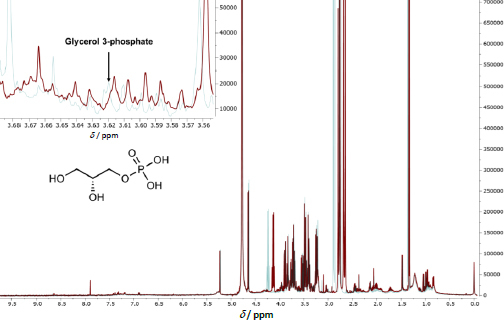
Results of NMR-Based Metabolomics Analysis. The 500 MHz ^1^H-NMR spectra of plasma obtained from patient 1 are presented. The spectral overlay reveals distinct differences between samples collected during the warfarin-mango interaction, when the INR was supratherapeutic (depicted in red), and those obtained after discontinuing mango consumption, when the INR stabilized within the therapeutic range (depicted in blue). A marked reduction in glycerol 3-phosphate concentration (at 3.62 ppm) during the warfarin-mango interaction is observed, as highlighted by the arrow

The 500 MHz ^1^H-NMR spectrum of the plasma sample obtained from patient 1 is illustrated in Figure 2. The chromatogram overlay highlights distinctions between the plasma samples collected during the warfarin-mango interaction and those obtained after mango discontinuation (control). Untargeted metabolomics analysis was used to screen the metabolites in plasma samples, and 208 metabolites were detected in both the warfarin interaction and control groups. Identification of metabolites was achieved by referencing the Human Metabolome Database [20].

The sparse partial least squares discriminant analysis (sPLS-DA) scores plot (Figure S1A) demonstrates a notable separation between the two groups: warfarin interaction (group 1, shown in red) and control (group 2, shown in green), indicating a distinct difference in their metabolic profiles. The loadings plot (Supplementary Material, Fig. S1B) illustrates the metabolites selected by the sPLS-DA model for each component, ranked by the absolute magnitude of their loadings. The top 10 metabolites are cyclic AMP, N-acetylaspartylglutamic acid, erythritol, N-methylhydantoin, N-acetyltaurine, 4-guanidinobutanoate, glycerol 3-phosphate, dopamine, L-3-hydroxykynurenine, and N'-formylkynurenine. multivariate exploratory receiver operating characteristic (ROC) analysis, which is based on cross-validation (CV) performance averaged across all models and CV runs (Figure S1C, D), assesses the diagnostic accuracy of the model. The area under the curve (AUC) of 0.904 indicates a strong ability to differentiate between the two groups (95 % confidence Interval [CI]: 0.729 to 1.000).

### Analysis of related pathways

**Figure 3.**
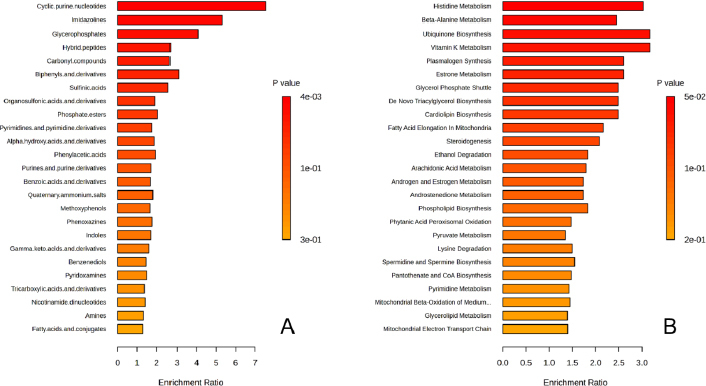
Pathway enrichment analysis mapping metabolites to specific metabolic pathways using (a) chemical structures and (b) the Small Molecule Pathway Database. The top 25 enriched metabolite sets are ranked in accordance with their Enrichment Ratio, based on chemical structures and SMPDB, respectively

Pathway enrichment analysis was conducted using MetaboAnalyst version 6.0 to map metabolites to specific pathways based on chemical structures and the Small Molecule Pathway Database (SMPDB). This analysis utilized 1,250 sub-class metabolite sets, including lipid-specific sets, and 99 metabolite sets aligned with normal human metabolic pathways. The top 25 enriched metabolite sets, shown in Figure 3A and 3B, were ranked by their Enrichment Ratio based on chemical structure and SMPDB, respectively. Metabolic disruptions were identified between the warfarin-mango interaction group and the control group. The pathways primarily affected amino acid metabolism, lipid metabolism, mitochondrial function, and cellular energy production. Key pathways altered include histidine metabolism, beta-alanine metabolism, ubiquinone biosynthesis, plasmalogen synthesis, estrone metabolism, the glycerol phosphate shuttle, *de novo* triglyceride biosynthesis, cardiolipin biosynthesis, fatty acid elongation in mitochondria, and steroidogenesis. These disruptions suggest that the warfarin-mango interaction may notably impair mitochondrial function and lipid metabolism, which may, in turn, influence cellular energy production. Such metabolic alterations could explain the observed increase in INR levels, indicating potential complications in the blood clotting cascade associated with this interaction.

**Figure 4.**
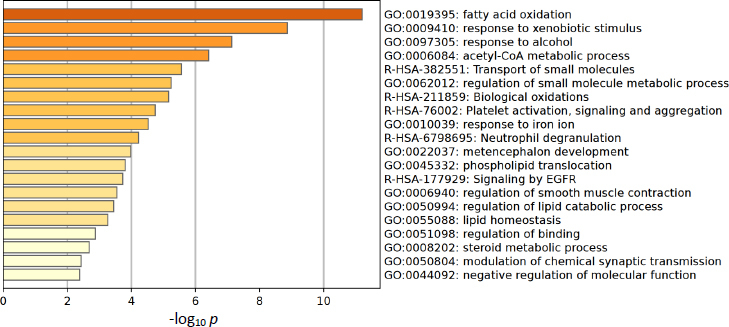
Pathway enrichment analysis bar chart illustrating the top 20 enrichment clusters, ranked by their -log10 (p) values, highlighting metabolic disruptions during warfarin-mango interactions

Metascape was employed to investigate pathway enrichment and clustering analysis to construct the metabolite-metabolite network. The top 20 enrichment clusters, shown in Figure 4, were ranked based on their -log_10_ *p* values.

Metabolic disruptions were observed in warfarin-mango interactions. The altered pathways identified in the enrichment analysis bar chart included fatty acid oxidation, response to xenobiotic stimulus, response to alcohol, acetyl-CoA metabolic process, transport of small molecules, regulation of small molecule metabolic processes, biological oxidations, platelet activation, signalling and aggregation, response to iron ion, and neutrophil degranulation.

**Figure 5.**
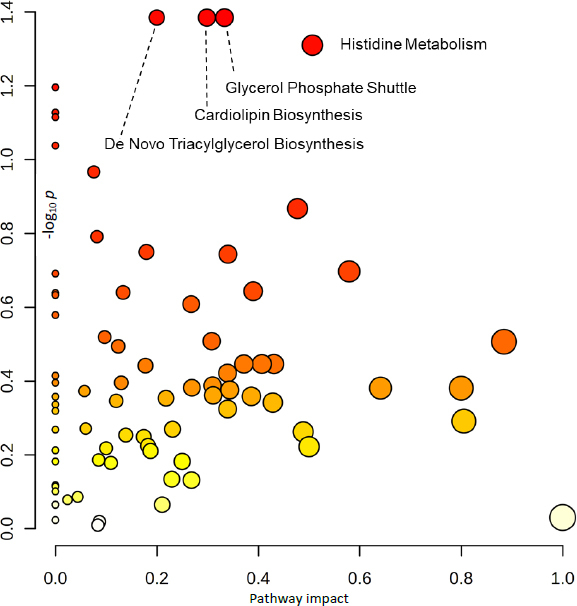
Pathway analysis results, based on -log10(p) values and pathway impact scores, identified four significantly disrupted metabolic pathways: histidine metabolism, glycerol phosphate shuttle, cardiolipin biosynthesis, and *de novo* triglyceride biosynthesis. These findings align with the results from the pathway enrichment analysis

**Figure 6.**
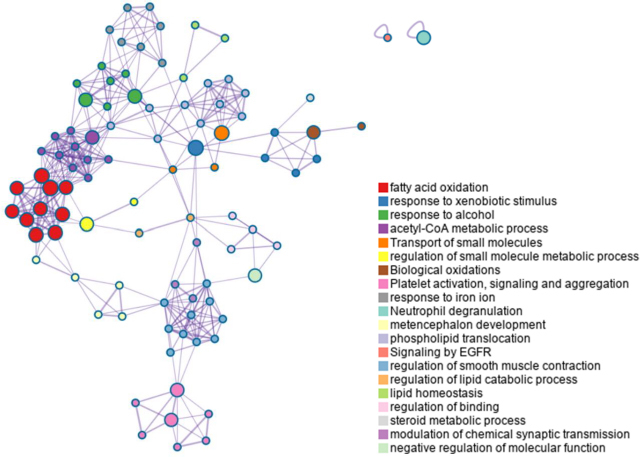
Metabolic network illustrating potential crosstalk between various pathways during the warfarin-mango interaction. The enrichment network visualization highlights both intra- and inter-cluster similarities among enriched terms, with up to ten terms per cluster, each uniquely color-coded for annotation

The pathway analysis results presented in Figure 5, conducted using MetaboAnalyst version 6.0, were based on -log_10_ *p* values and pathway impact scores. The analysis identified four significantly disrupted metabolic pathways: histidine metabolism, glycerol phosphate shuttle, cardiolipin biosynthesis, and *de novo* triglyceride biosynthesis, which are all consistent with the pathway enrichment findings. Subsequently, we used Metascape to construct a metabolic network aimed at elucidating potential crosstalk among these pathways during the interaction period between warfarin and mango. The Metascape enrichment network visualization further highlights intra- and inter-cluster similarities of enriched terms, with up to ten terms per cluster, each color-coded for annotation, as shown in Figure 6. This methodological approach may facilitate the identification of key novel metabolic pathways, thereby enhancing our understanding of metabolic regulation during the warfarin-mango interaction. These findings emphasize the importance of understanding dietary interactions with medicine to ensure safe and effective therapeutic outcomes.

### Alterations in metabolic profiles during warfarin-mango interaction

**Figure 7.**
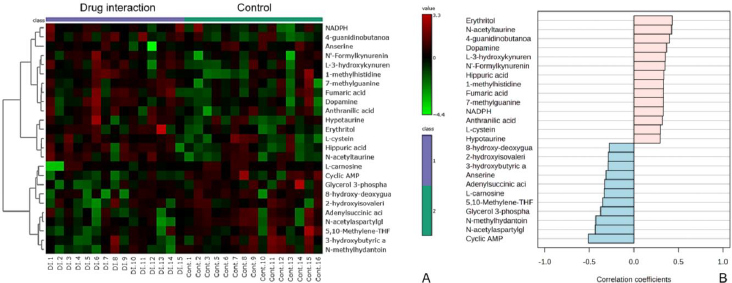
(a) Heatmaps illustrating the differential abundance of metabolites between the warfarin-mango interaction and control data, highlighting metabolites that display increasing or decreasing trends during the interaction. In these heatmaps, columns represent individual samples divided into two groups: the warfarin-mango interaction and the control data, while rows display the top 25 metabolites ranked by their correlation coefficients. Normalized metabolite intensities are visualized using a colour scale ranging from red to green, where red signifies higher intensities and green represents lower intensities. (b) Bar graph depicting metabolic alterations, with Pearson correlation coefficients calculated to assess the relationships among metabolites. This analysis reveals distinct patterns during the warfarin-mango interaction, which are consistent with the trends observed in the heatmap

The heatmaps (Figure 7A) illustrate the differential abundance of metabolites between the warfarin-mango interaction and control data, highlighting metabolites that exhibit either increasing or decreasing trends during the interaction.

In these heatmaps, columns represent individual samples, divided into two groups: the warfarin-mango interaction and control, while rows display the top 25 metabolites, ranked by their correlation coefficients. The normalized metabolite intensities are visualized using a colour scale ranging from red to blue, where red signifies higher intensities and blue represents lower intensities.

To further investigate the metabolic alterations, Pearson correlation coefficients were calculated to assess the relationships among metabolites, revealing distinct patterns during the warfarin-mango interaction. These correlations are depicted in the bar graph (Figure 7B), which aligns with the trends observed in the heatmap. Collectively, the data suggest significant alterations in metabolite profiles, indicating that the warfarin-mango interaction induces notable metabolic changes.

### Key metabolites highlighted as a potential biomarker

**Figure 8.**
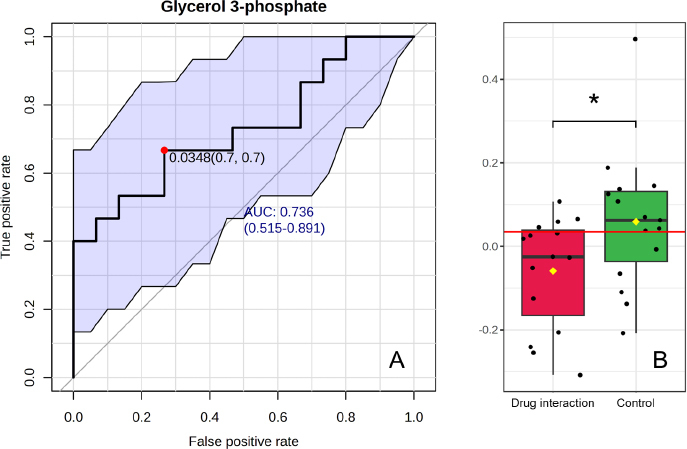
(a) The ROC curve for glycerol 3-phosphate, demonstrating an area under the ROC curve (AUROC) of 0.72889, suggesting its potential as a reliable biomarker (p = 0.041216). (b) Comparison of glycerol 3-phosphate concentrations during the warfarin-mango interaction (red) versus after discontinuing mango consumption (control, green), indicating a significant reduction in glycerol 3-phosphate during the drug interaction

Univariate receiver operating characteristic (ROC) curve analyses were performed to evaluate the area under the ROC curve (AUROC) and calculate the 95 % confidence intervals for individual biomarkers. The results of the ROC curve analysis ranked the biomarkers based on their AUROC, T-statistics, and log_2_ fold change (FC), as shown in Table 2. Key metabolites were selected based on an AUC value of 0.7 and statistically significant differences between the two groups: warfarin-mango interaction and control. Eight key metabolites were identified, including N-acetylaspartylglutamic acid, cyclic AMP, erythritol, N-methylhydantoin, 4-guanidinobutanoate, glycerol 3-phosphate, N-acetyltaurine, and dopamine. Among these, glycerol 3-phosphate was chosen as a potential biomarker to elucidate the metabolic alterations occurring during the warfarin-mango interaction. Glycerol 3-phosphate is involved in key metabolic pathways, including the glycerol phosphate shuttle, cardiolipin biosynthesis, and *de novo* triglyceride biosynthesis, findings which align with those from pathway enrichment analyses. The ROC curve for glycerol 3-phosphate is presented in Figure 8, with an AUROC of 0.72889, indicating its potential as a good biomarker (*p* = 0.041216).

**Table 2.** Receiver operating characteristic (ROC) curve analysis of individual biomarkers

Biomarkers	AUROC	*T*-test	log_2_ FC
N-acetylaspartylglutamic acid	0.81778	0.016876	-0.23413
Cyclic AMP	0.80000	0.004005	-0.54444
Erythritol	0.78222	0.017716	0.54163
N-methylhydantoin	0.73333	0.018364	-0.15266
4-guanidinobutanoate	0.72889	0.027884	0.22892
Glycerol 3-phosphate	0.72889	0.041216	-0.49244
N-acetyltaurine	0.72000	0.019383	0.5282
Dopamine	0.71111	0.045276	0.31138

AUROC = area under ROC curve; FC = fold change

**Figure 9.**
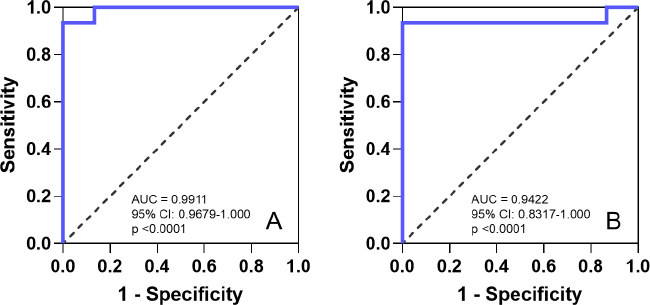
Receiver Operating Characteristic (ROC) curve analyses enable assessment of the area under the curve (AUC) and determine the 95 % confidence intervals for the International Normalized Ratio (INR) values in comparison to glycerol 3-phosphate concentrations during the warfarin-mango interaction and after mango discontinuation (control). (a) The AUC during the warfarin-mango interaction was 0.9911 (95 % CI: 0.9679 to1.000). (b) The AUC following mango discontinuation (control) was 0.9422 (95 % CI: 0.8317 to 1.000). This further supports the potential of glycerol 3-phosphate as a reliable biomarker

ROC curve analyses were then conducted to assess the AUC and determine the 95 % confidence intervals for the INR values in comparison to glycerol 3-phosphate concentrations during the warfarin-mango interaction and following mango discontinuation (control), as shown in Figure 9A and 9B, respectively. The AUCs were 0.9911 (95 % CI: 0.9679 to 1.000) and 0.9422 (95 % CI: 0.8317 to 1.000), respectively, further confirming the potential of glycerol 3-phosphate as a reliable biomarker.

## Discussion

In our study, all patients experienced an increase in INR following mango consumption. As soon as the consumption of mango was identified as a possible cause of the supratherapeutic INR, the patients were advised to discontinue it. Subsequently, the mean INR decreased from 4.57 ± 1.46 to 2.45 ± 0.39, with a statistically significant difference in INR levels between periods of mango ingestion and post-discontinuation (*p* <0.0001). These results suggest that mango consumption may influence the of warfarin, a finding which is in alignment with previous studies [5,7]. However, the mechanisms underlying this drug interaction remain poorly understood [5,7]. Therefore, this study aimed to further elucidate the interaction between warfarin and mango by integrating clinical findings with molecular insights derived from ^1^H-NMR analysis.

Metabolomic analysis identified glycerol 3-phosphate as a potential biomarker for elucidating the metabolic alterations associated with the warfarin-mango interaction. Glycerol 3-phosphate plays a critical role in several metabolic pathways, including the glycerol phosphate shuttle, cardiolipin biosynthesis, and *de novo* triglyceride biosynthesis, which were highlighted in our pathway enrichment analyses. The enrichment analysis and metabolite-metabolite network from Metascape also revealed a connection to platelet activation signalling and aggregation. Additionally, ROC curve analyses were performed to evaluate the AUC of INR values in relation to glycerol 3-phosphate concentrations, further supporting its potential as a reliable biomarker. Our findings demonstrated a decrease in the concentration of glycerol 3-phosphate during the warfarin-mango interaction, consistent with a previous study that reported a significant increase in glycerol 3-phosphate concentrations during platelet activation [23]. This inverse relationship suggests that the mechanism underlying this interaction may be attributable to interference with platelet metabolism associated with alterations in glycerol 3-phosphate concentrations. Additionally, a previous study suggests that glycolysis serves as a major connecting link between various metabolic pathways in platelets [23]. Glycolytic intermediates produced during metabolic switching facilitate lipid, nucleotide, and amino acid metabolism, thereby supporting platelet activation [23]. Notably, glycerol 3-phosphate, a key metabolite, promotes the synthesis of other cellular phospholipids essential for platelet activation [23].

Platelet activation is a highly dynamic process characterized by the activation of integrin αIIbβ3, degranulation, aggregation, and cytoskeletal rearrangement [23-25]. These events arise from a synergistic interplay between platelet signalling and metabolic pathways [23-25]. In their resting state, platelets rely on both glycolysis and oxidative phosphorylation to meet their energy requirements [23,26]. Although activated platelets preferentially shift their energy metabolism towards glycolysis, mitochondrial oxidation remains essential, effectively compensating for any impairment in glycolysis and ensuring adequate energy supply [23,27-32]. Recent studies suggest that targeting specific metabolic pathways in platelets may provide therapeutic strategies to mitigate platelet activation and thrombosis [27,28,31,33]. Platelet activation is known to be associated with enhanced aerobic glycolysis, and this process, along with the key substrates derived from one-carbon metabolism, plays an important role in facilitating platelet activation [23].

**Figure 10.**
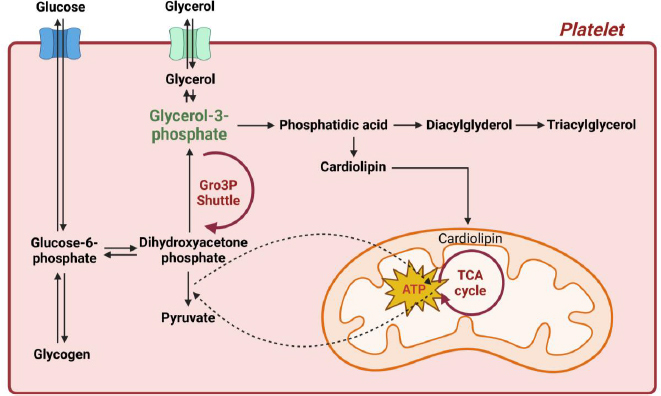
Proposed mechanism of warfarin and mango interaction interconnected through glycerol-3-phosphate via the glycerol phosphate shuttle, cardiolipin biosynthesis, and *de novo* triglyceride biosynthesis. The decrease in glycerol-3-phosphate may impair energy production in glycolysis, potentially disrupting platelet function and contributing to the observed increase in INR levels in all patients

Glycerol-3-phosphate is a central metabolite intersecting four critical pathways in cellular metabolism: 1) glycolysis, 2) glycerolipid synthesis and the glycerolipid/free fatty acid cycle, 3) gluconeogenesis, and 4) energy metabolism via the glycerol phosphate shuttle, which facilitates electron transfer to mitochondria [34]. Cytoplasmic glycerol-3-phosphate dehydrogenase (cG3PDH) reduces dihydroxyacetone phosphate (DHAP) to glycerol-3-phosphate, utilizing NADH [34-37]. Glycerol-3-phosphate is subsequently oxidized back to DHAP by mitochondrial glycerol-3-phosphate dehydrogenase (mG3PDH), transferring electrons to FAD to generate FADH_2_, which enters the electron transport chain for oxidative phosphorylation [34-37]. This process effectively transfers electrons from cytosolic NADH to mitochondrial FAD^+^, contributing to ATP production [34-37]. Concurrently, glycerol-3-phosphate serves as a precursor for cardiolipin biosynthesis, an essential phospholipid that constitutes a significant portion of the inner mitochondrial membrane [38-43]. This pathway involves multiple enzymatic steps, starting with the formation of glycerol-3-phosphate, followed by acylation reactions that lead to cardiolipin production [38-43]. This synthesis pathway is essential for mitochondrial function and the integrity of the electron transport chain [38-43]. Additionally, *de novo* triglyceride biosynthesis begins with glycerol-3-phosphate derived from glycolysis, which is then processed enzymatically to form triacylglycerol, a key energy storage molecule [44-47]. Together, the glycerol phosphate shuttle, cardiolipin biosynthesis, and *de novo* triglyceride biosynthesis are interconnected through glycerol-3-phosphate, highlighting its essential role in cellular metabolism, including platelet activation (Figure 10).

To date, this study is the first to employ ^1^H-NMR metabolomics analysis to elucidate the mechanisms of drug interaction, integrating clinical findings with molecular insights. Our results suggest specific molecular mechanisms through which the warfarin-mango interaction disrupts platelet energy metabolism, with glycerol 3-phosphate identified as a potential biomarker. Our findings highlight that the decrease in glycerol 3-phosphate may impair energy production in glycolysis, potentially disrupting platelet function and contributing to the observable increase in INR levels in all patients. Furthermore, this interaction may synergistically influence the clearance of warfarin, as mango inhibits CYP450 enzymes. However, further investigations are essential to clarify the precise pathways involved. Future studies should include larger sample sizes and be conducted across multiple centres to enhance generalizability. The application of ^1^H-NMR metabolomic analysis holds promise for providing valuable insights into these interactions, as demonstrated by the success of previous investigations [16].

## Conclusions

This study is the first to employ ^1^H-NMR metabolomics analysis to investigate the molecular mechanisms underlying drug interactions, specifically focusing on the warfarin-mango interaction. By integrating clinical outcomes with molecular insights, our findings suggest that this interaction disrupts platelet energy metabolism, with glycerol 3-phosphate emerging as a potential biomarker. The reduction in glycerol 3-phosphate may disrupt energy production through glycolysis, potentially affecting platelet activation and contributing to the elevated INR levels observed in all patients. The use of ^1^H-NMR to monitor metabolites during the interaction between warfarin and mango presents a promising approach for gaining a more comprehensive understanding of such drug interactions.

## Supplementary material

Additional data are available at https://pub.iapchem.org/ojs/index.php/admet/article/view/2740, or from the corresponding author on request.


