# Supplementary material for: Metabolic insights into the warfarin-mango interaction: A pilot study integrating clinical observations and metabolomics
Source: ADMET DMPK. 2025 Jun 8;13(3):2740. doi: 10.5599/admet.2740 (PMC12205924; doi:10.5599/admet.2740)
Supplement: Supplementary file 1 [file ADMET-13-2740-S1.pdf]

Open Access : ISSN : 1848-7718

http://www.pub.iapchem.org/ojs/index.php/admet/index

Supplementary material to

Metabolic insights into the warfarin-mango interaction: a pilot study integrating clinical observations and metabolomics

Piyapat Rattanasuwan[1], Prem Lertpongpipat[1], Natthapat Hiranchatchawal[1], Konwalin Wannaphueak[1], Sakonwan Pounghom[2], Parinya Thongkhao-on[2], Matchuda Suwanthai[2], Duangthip Sompradee[2], Auiporn Saithongdee[2], Churdsak Jaikang[3,4] and Preechaya Tajai[3,4]

[1]*Faculty of Medicine, Chiang Mai University, Chiang Mai 50200, Thailand*
[2]*Hua Hin Hospital, Prachuap Khiri Khan 77110, Thailand*
[3]*Department of Forensic Medicine, Faculty of Medicine, Chiang Mai University, Chiang Mai, 50200, Thailand*
[4]*Metabolomic Research Group for Forensic Medicine and Toxicology, Department of Forensic Medicine, Faculty of Medicine, Chiang Mai University, Chiang Mai 50200, Thailand*

ADMET & DMPK **13(3)** (2025) 2740; https://doi.org/10.5599/admet.2740

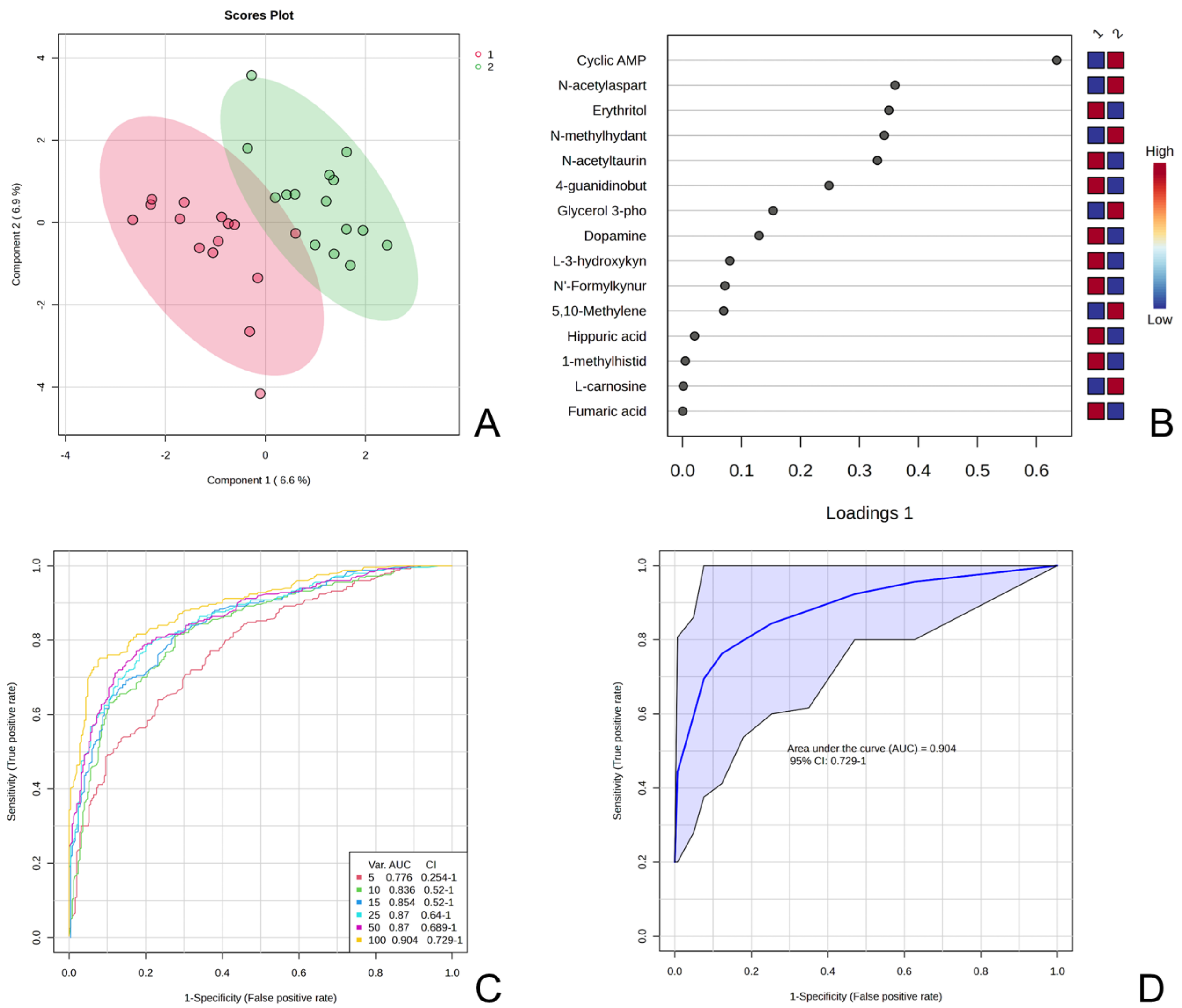

Figure S1. (a) The Sparse Partial Least Squares Discriminant Analysis (sPLS-DA) scores plot shows a notable separation between the two groups: warfarin interaction (group 1, shown in red) and control (group 2, shown in green). (b) The loadings plot illustrates the metabolites selected by the sPLS-DA model for each component, ranked by the absolute magnitude of their loadings. (c) Multivariate Exploratory Receiver Operating Characteristic (ROC) Analysis, which is based on cross-validation (CV) performance averaged across all models and CV runs, assesses the model’s diagnostic accuracy. (d) The area under the curve (AUC) of 0.904 indicates a strong ability to differentiate between the two groups (95% Confidence Interval [CI]: 0.729 to 1.000)

cc BY
