# Supplementary material for: Metabolic insights into the warfarin-mango interaction: A pilot study integrating clinical observations and metabolomics
Source: ADMET DMPK. 2025 Jun 8;13(3):2740. doi: 10.5599/admet.2740 (PMC12205924; doi:10.5599/admet.2740)
Supplement: Supplementary file 2 [file ADMET-13-2740-S1.docx]

*ADMET & DMPK 13(3) (2025) S2740*

*
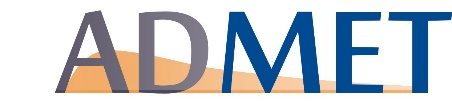
***Open Access : ISSN : 1848-7718**[***http://www.pub.iapchem.org/ojs/index.php/admet/index***](http://www.pub.iapchem.org/ojs/index.php/admet/index)

Supplementary material to

**Metabolic insights into the warfarin-mango interaction: a pilot study integrating clinical observations and metabolomics**

Piyapat Rattanasuwan^1^, Prem Lertpongpipat^1^, Natthapat Hiranchatchawal^1^, Konwalin Wannaphueak^1^, Sakonwan Pounghom^2^, Parinya Thongkhao-on^2^,
Matchuda Suwanthai^2^, Duangthip Sompradee^2^, Auiporn Saithongdee^2^,
Churdsak Jaikang^3,4^ and Preechaya Tajai^3,4^

^1^Faculty of Medicine, Chiang Mai University, Chiang Mai 50200, Thailand
^2^Hua Hin Hospital, Prachuap Khiri Khan 77110, Thailand
^3^Department of Forensic Medicine, Faculty of Medicine, Chiang Mai University, Chiang Mai, 50200, Thailand
^4^Metabolomic Research Group for Forensic Medicine and Toxicology, Department of Forensic Medicine, Faculty of Medicine, Chiang Mai University, Chiang Mai 50200, Thailand

ADMET & DMPK **13(3)** (2025) 2740; <https://doi.org/10.5599/admet.2740>


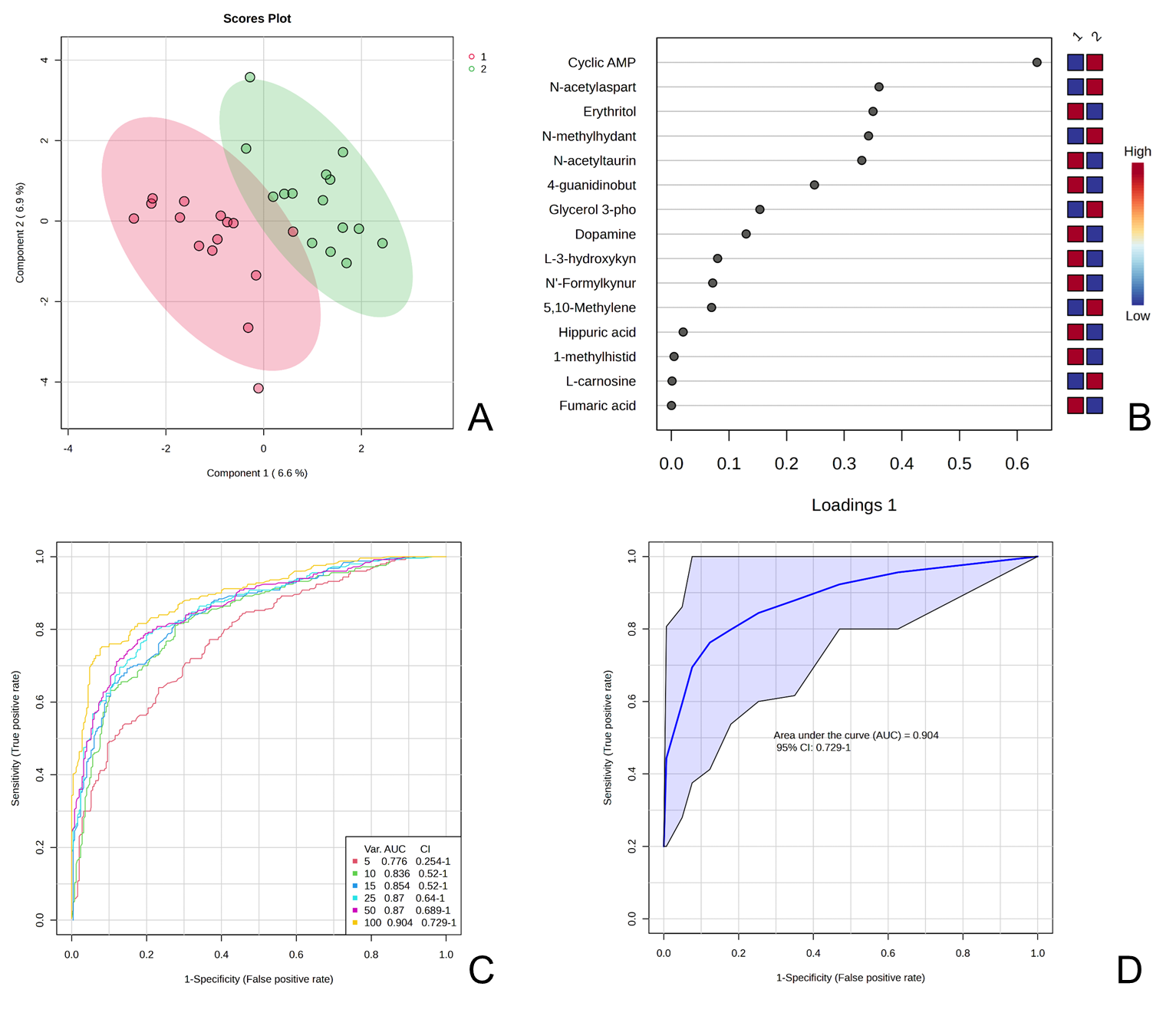


**Figure S1.** (a) The Sparse Partial Least Squares Discriminant Analysis (sPLS-DA) scores plot shows a notable separation between the two groups: warfarin interaction (group 1, shown in red) and control (group 2, shown in green). (b) The loadings plot illustrates the metabolites selected by the sPLS-DA model for each compo­nent, ranked by the absolute magnitude of their loadings. (c) Multivariate Exploratory Receiver Operating Characteristic (ROC) Analysis, which is based on cross-validation (CV) performance averaged across all models and CV runs, assesses the model’s diagnostic accuracy. (d) The area under the curve (AUC) of 0.904 indicates a strong ability to differentiate between the two groups (95% Confidence Interval [CI]: 0.729 to 1.000)
